# Salivary glands and lymph nodes

**DOI:** 10.1186/1470-7330-15-S1-O25

**Published:** 2015-10-02

**Authors:** Harriet C Thoeny

**Affiliations:** 1 University Hospital of Bern, Bern, Switzerland

## 

Involvement of lymph nodes in various pathologies of the head and neck is frequent not only in malignant diseases but also in inflammatory conditions. As size criteria are not sufficient to define a lymph node as malignant, other criteria such as shape, central necrosis and extracapsular spread are other helpful signs. However, micrometastases are still an unresolved problem although new imaging methods have already shown promising results. Typical imaging features of CT and MRI indicating extracapsular spread and carotid artery invasion will be discussed as these have therapeutic and prognostic implications.

Although salivary gland pathologies are relatively rare, its large variety of differential diagnoses makes it challenging. In children and pregnant women, sonography is the first step, and CT is the method of choice in inflammatory disease. MRI is the first line examination in palpable salivary gland masses to assess the exact extent of tumours, the invasion of neighbouring structures, perineural spread and bone invasion. Differential diagnoses and imaging features of the most frequent tumour types will be discussed and an approach to differentiate between benign and malignant lesions will be provided.

